# Phase transitions, collective emotions and decision-making problem in heterogeneous social systems

**DOI:** 10.1038/s41598-019-54296-7

**Published:** 2019-12-02

**Authors:** Dmitriy Tsarev, Anastasiia Trofimova, Alexander Alodjants, Andrei Khrennikov

**Affiliations:** 1 ITMO University, Laboratory of Quantum Cognition and Intelligent Systems, St. Petersburg, 197101 Russia; 2 ITMO University, Faculty of Laser Photonics and Optoelectronics, St. Petersburg, 197101 Russia; 3 Linnaeus University, International Center for Mathematical Modeling in Physics and Cognitive Sciences, Växjö, SE-351 95 Sweden

**Keywords:** Bose-Einstein condensates, Human behaviour

## Abstract

The paper considers the problem of collective decision-making as a second order phase-transition, which occurs in heterogeneous information-oriented communities possessing frequent information exchange between individuals. We examine the quantum-like model of simplified two-level cognitive systems (TLCS) interacting with a socially important (contextual) information field. The model exploits approaches to the modern social cohesion framework. We refer to some target network community, which is in close interaction (e.g. message exchange) with “reservour” (large network community) possessing infinite degree of freedom. We introduce a new approach for valence and arousal variables, used in cognitive sciences for the description of collective emotion states. We express them via collective polarization and population imbalance respectively. The model predicts a super-radiant phase transition for target network community leading to coherent polarization establishment in the socium. The valence and arousal parameters can be evaluated from actrors behaviour in social network communities as a result of immediate response (decision-making) to some notable news. We introduce Gaussian and Levy distribution functions to reveal the influence of social community inhomogeneity on decision-making features. We show that a critical (social) temperature is determined by the population imbalance (valence), detuning, field coupling strength parameter and relay to conditions of social polarization establishment. We predict coherent social energy release in a community without inversion due to its specific properties close to the superfluid paradigm in quantum physics, or social cohesion in sociology. We establish a connection of our model to the recently presented quantum-like model of the social laser to describe the waves of social protests as the result of collective decision-making process in the system with inversion of population in mental states. Finally, we compare our model with existing social impact models, a.k.a. cellular automata models, in the limit when social community perceives information field in the state induced by large information reservoir (mass-media). Notably, eliminating quantized field we lose important information how collective emotions (arousal) form in social community.

## Introduction

Nowadays, elaboration of appropriate quantum-like physical models in psychology, decision-making, and social science evokes rapidly growing interest (monographs^1–5^, see also some representative papers^6–15^). The applications of the mathematical formalism of quantum mechanics, especially quantum information and the probability calculus, outside physics can be considered as one of the fruits of the quantum information revolution (maybe the unexpected one). The new viewpoint on the quantum theory based on the *information interpretation* (see, e.g.^16–22^) stimulated applications to psychology, cognition, decision-making, and sociology. Humans are treated as information transformers. Moreover there is a plenty of experimental data collected in these areas^6–8,11^, which is naturally described by operating with complex probability amplitudes, instead of classical probability measures. Various approaches are used for the formulation of a quantum like model for decision-making problem. In particular, they exploit the calculus of quantum operator and methods of quantum field theory^23,24^, quantum measurement paradigm^25–27^, quantum version of a classical (dynamical) Markov model^28^, quantum-like Bayesian Network formalism^29,30^. We emphasize the role of Quantum Bayesianism (QBism)^18,22^ which highlights the possibility of the subjective interpretation of quantum probabilities and the role of individual agents making decisions on the basis of the probabilistic rules of quantum theory. Notably, applications of quantum-like modeling in psychology and decision-making demonstrate its efficiency and relevance to some real life problems in comparison with classical (probabilistic) models^1–5,7–15,23,24^. We remark that these studies are closely connected to the research on irrationality (violation of the Sure Thinking Principle, cf.^31^) and applicability of the classical probability laws in decision-making (the works of Kahenman, Tversky, Shafir^32–35^).

In the paper we argue that computer-mediated communication between people is inherent to information-oriented societies and creates new realities in our life, dealing with socially actual information transmission and exchange. They require kindly new approaches for accurate description and characterization of collective behaviors and emotional states in modern social and behavioral sciences^4,36,37^. By mapping social network problems onto physical models one can obtain a powerful practical toolset to solve current problems of collective decision-making, appearing at the border of natural and social sciences, psychology, and decision theory. In fact, we speak here about a new area of implementing many-body quantum physics formalism for complex psychosocial processes simulation^38^. Up to now for these purposes researchers performed social computation experiments based on various social network models in the framework of game theory^39^.

However, social systems possess their own specifics that arise from behavioral features of individuals in decision-making processes^1,2^.

As it was shown many years ago, two-level (spin) systems are suitable for modeling social impact phenomena introduced by Latane in^40^ and then approved in sociological studies^41,42^. Notably, in the middle of 1990s the social impact problem allowed to create a new class of cellular automata models, where Ising-type interaction was assumed^43–46^. The models proposed enabled taking into account the combination of several types of social influences, such as behavioral contagion, community pressure, social facilitation, etc., known in sociology^40–42^. The concepts of “social space” and “social distance” play an essential role in such models^45,46^.

However, actual psychological and sociological studies stress the importance of contextual information fields influences on individuals due to various current technological facilities for communication^47,48^. Obviously, variation of agents’ emotional states in the network represents a natural result of such influences^49^. Although emotions individuals exhibit may be not so simple in real world, the emotional states can be effectively represented in a two dimensional Arousal - Valence space, as it was firstly introduced by Russell in the framework of circumplex model of affect^50^. Further studies aimed at measuring the emotional states of individuals only confirmed this model, see e.g.^51–53^. In particular, Schweitzer and Garcia in^37^ suggested agent-based arousal - valence modelling of collective emotions by using classical Brownian particle properties.

The paper considers the possible influence of the information field carrying some context on the community through changes in emotional and cognitive states of individuals^49^. In particular, such an influence can be described in the framework of quantum-like modeling, like that recently presented to describe human behavior and decision-making problems^2^. To be more specific in the paper we are interested in coherent effects appearing various emotional states formation cf.^51–53^. In modern sociology “coherence” could manifests itself as some social group cohesion exhibiting collective decision-making phenomena cf.^54–56^.

In the paper we use physical approach to coherence phenomena, which is well known in laser physics. We remind that Haken, one of the fathers of laser physics^57^, already discussed the possibility of laser functioning formalism and phase-transition concept application to the process of decision-making in social systems in the framework of Synergetics^58^. Recently, Weidlich and Haag applied statistical approach to characterize the opinion formation and related topics in sociology^59^.

There are a lot of real life examples when collective decision-making appears. We refer here to the process of a collective opinion formation representing “macroscopic” coherent phenomena^45,46^. Another important example characterizing collective decision-making in a specific social context is so-called social lasing effect recently introduced to explain a social energy release by a crowd as “an ensemble” of collectively excited individuals^60^.

It is important to stress that computer-mediated communication between people plays crucial role in social communities exhibiting various “coherence” features. In particular, information cascade processes occurring in social networks nowadays represent an indispensable tool for influence maximization in real-world social systems^61–63^. For instance, in the case of social laser the “lower” and “upper” levels (of TLCSs) can be recognized as passive (non-ready to actions) and active (ready to actions) quantum-like states of individuals who can be involved in protest actions. In connection with the social laser paradigm information cascades allow to form (within a historically short time period) the so-called “population inversion” in individuals’ mental state that, midly speaking, represents a broad protest community. In common lasers the lasing effect starts from spontaneous emission when the pump is just above the threshold. In terms of social laser, spontaneous emission may be recognized as spontaneous decision-making of some individuals that gives rise to a collective decision-making processes accompanied with occupation of ground (non-excited) state. Thus, in this case the cascade model leads to the amplification of social protests when time delay between two or more protest actions vanishes. In general, cascade models are relevant to Mark Granovetter’s familiar threshold model of collective behavior^64^.

Although a laser model looks quite attractive for sociologists, it requires some further specifications and deeper understanding in the framework of collective decision-making problem. We ask here a vital question: is it possible to achieve coherent social energy release in a social system without inversion, i.e. without its high excitation?

To answer the question, we refer here to a so-called bosonic simulation effect that looks more general and occurs in various systems including lasers and those exhibiting phase-transition properties^65^.

The term “laser” in quantum technologies was used to describe a bosonic device, such as optical^66^, atomic^67^, polaritonic^68^, plasmonic^69^, etc. This device produces coherent state of photons or matter-field and requires no population inversion. Strictly speaking such devices are not lasers according to the definition given in textbooks^65^. Moreover, the rigorous description of these systems may be established by using a thermodynamic approach; the temperature is a macroscopic parameter characterizing thermodynamic features of the whole system being in thermal equilibrium.

Bose-Einstein condensation (BEC) phenomenon, representing the second order phase-transition, manifests coherent properties of such “lasers” when macroscopically large number of particles occupies a single ground quantum state of energy at temperature below critical^70,71^. Noteworthy, social networks in the limit of “winner-takes-all” exhibit BEC phenomenon. In particular, within the statistical physics approach some networks admit mapping onto equilibrium Bose gas, which possesses a condensation effect when the single (“quantum”) node overtakes a large number of links^72^. Despite the fact that the current theories based on the statistical physics approach predict qualitatively true results for dynamically growing social networks, the determination and measurement of the critical temperature represents an actual problem for this approach. It is important that the definition of the temperature parameter in social sciences is contextual and requires additional specifications.

The concept of social temperature we use in the paper may be interpreted as a parameter that characterizes the degree of randomness (large scale fluctuations) in individuals’ behavior evoked by a number of socio-economic reasons^73^. These sometimes unwanted fluctuations can be suppressed in the so-called “low temperature” limit when thermal energy is much smaller than other characteristic energy scales. In quantum statistical physics BEC concept is close to superfluid properties occurring due to weak interaction between the particles^70^. Superfluidity characterizes the capability of quantum liquid to flow without losses, with zero viscosity. In respect to social systems we can recognize social superfluidity as an opportunity for humans to fully realize their collective decision-making coherently, without any “frictions” and resistance. In fact, superfluid properties of some community are defined by its cohesion^55^.

In this paper we offer superradiant phase-transition (SPT) paradigm known in quantum optics to characterize a collective decision-making problem and emotion formation in network community in the presence of quantum-like mass-media influence, cf.^74–81^. We exploit the concept of “social atom” that dates back to William Adams’ work^82^. At the beginning of 21th century the concept of social atom as “elementary building block of the social world” appeared in the works of Mark Buchanan^83^, Serge Galam, Andrei Khrennikov, and others who attempted to describe social and crowd behavior in the framework of sociophysics approach^84^. In particular, the concept of a social atom is useful if we apply statistical physics approach to describe social dynamics and phase-transitions occurring in various network systems^85^. Remarkably, in real life an individual’s cognitive structure is rich and the number of mental energy levels may be infinite in general. However, individuals make some decisions in a contextual and probabilistic way determined by certain events, which previously happened in real or virtual life. To be more specific we restrict ourselves introducing a simple two-level cognitive system (TLCS) model that characterizes the features of s-atom in some context possessing discrete social or mental energy states. Such systems presume the absorption and emission of s (social)-photons characterizing quanta of socially actual “information” similar to “usual” two-level atoms in quantum physics^65^. Noticing, that s-photons carrying s-information have nothing common with the photons which can be measured in physical experiments. In contrast to routine Shannon’s information approach the s-information that we discuss in the paper is meaningful only in terms of a human decision-making problem. Shannon in^86^ underlines that “the semantic aspects of communication are irrelevant to the engineering aspects”. However, the semantic aspect, which is closely connected with contextual and emotional features of messages, plays an essential role in computer-oriented communication between the actors. The quantum approach to information retrieval problem should be mentioned as well^87^.

It worth noticing that some other quantum approaches which explore quantum Bayesian view of probabilities for mental state description can bring us to some different interpretations of Shannon information and its role in cognitive science, cf.^88^. However, this question is out of scope of this paper.

The paper is arranged as follows. In Sec. 1 we establish SPT problem taking into account heterogeneous peculiarities of human community consisting of individuals in terms of the decision-making problem^64^. To be more specific, we analyse this problem examining some (target) network community that we take as a part of a large social network. Our model of coupled photon-TLCS states, inherent to the target network, enables to characterize collective emotions in the presence of s-field. We treat social information sources as quantum-like ones producing s-photons and capable to change individuals’ mental states and collective emotions in a probabilistic way^49,60^. In Sec. 2 we analyse some crucial properties of SPT in connection with social behaviour. In particular, our model can describe processes occurring in the information oriented societies and is close to information prosumer concept for individuals. Modern computer-mediated communication between humans enforces them to produce and absorb some content representing s-photons in terms of sociophysics. The interaction between prosumers leads to social energy “equilibration” or, more rigorously, thermalization of social system as it happens with common atoms in the presence of collisions^78^. As we will see below, the phase-transitions, and especially SPT, occurring in such systems allow to release coherent social energy, and to create new phases without inversion in social systems, i.e. without social protests. This phases characterise some definite polarization and may be understood through social cohesion. In Sec. 3 we establish the connection of our model with social impact models considered before. In Sec. 4 we summarize our results.

## Physical Approach to Collective Decision-Making Problem

### Basic (quantum) properties of social systems interacting with social information field

**Figure 1:**
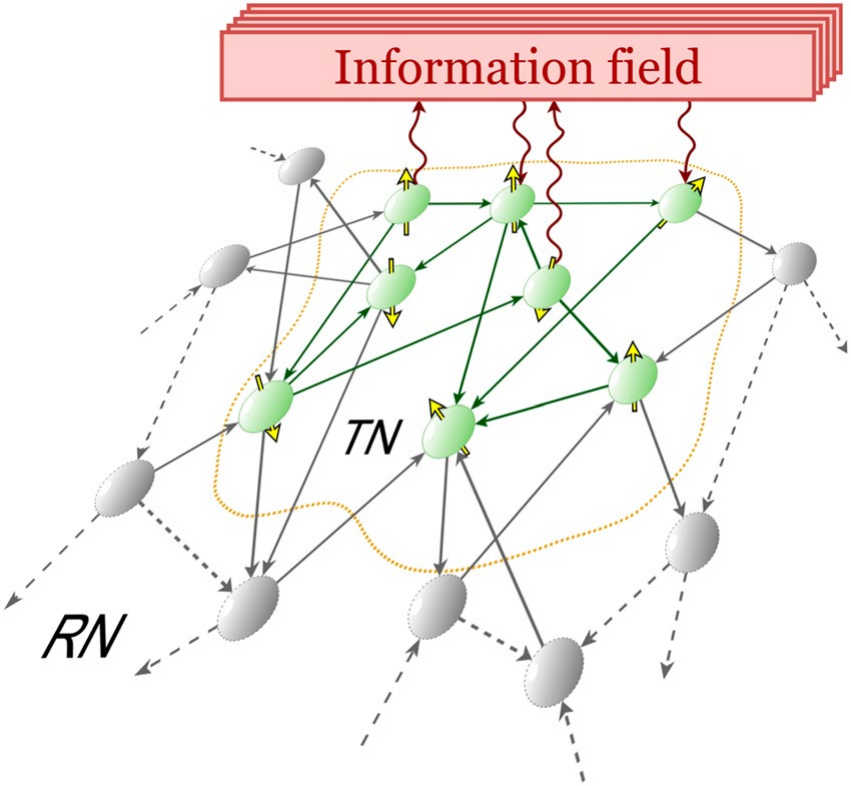
Sketch of social network (graph) coupled with information field. According to grand-canonical thermodynamic approach the total system consists of target network (TN) of *N* actors (green color vertices), information field subsystem and the “reservour”, RN (grey color vertices). Actors represent TLCSs and are established by yellow arrows indicating own “spin” directions. Graph links demonstrate information field transfer.

To introduce the model desired we use a common second quantization formalism based on Hamiltonian approach. We consider the interaction of social community (that we call target network) containing *N* TLCSs (s-atoms) with a quantum single mode (external) s-field described by annihilation (creation) operator *f*(*f*^†^)^78^, see Fig. 1. We describe such a system with the Hamiltonian

1 $$H=\hslash {\omega }_{{\rm{ph}}}{f}^{\dagger }f+\hslash \,\mathop{\sum }\limits_{j}^{N}\,\frac{{\omega }_{{\rm{at}},j}}{2}({b}_{j}^{\dagger }{b}_{j}-{a}_{j}^{\dagger }{a}_{j}))+\frac{\hslash }{\sqrt{N}}\,\mathop{\sum }\limits_{j}^{N}\,{g}_{j}({f}^{\dagger }{a}_{j}^{\dagger }{b}_{j}+{b}_{j}^{\dagger }{a}_{j}f),$$

where bosonic operators ${a}_{j}({a}_{j}^{\dagger })$ and ${b}_{j}({b}_{j}^{\dagger })$ describe annihilation (creation) of *j*-th TLCS decision at the lower $(|a\rangle )$ and upper (excited, $|b\rangle$) mental state, correspondingly; *g*_*j*_ is the coupling coefficient; ${\omega }_{{\rm{ph}}}$ characterizes frequency (energy) of s-field, ${\omega }_{{\rm{at}},j}$ is a frequency space for TLCSs. Thereafter we remove “s” letter for brevity and suppose $\hslash =1$ for our calculus. Thus, in our discussions the energy of something is given in frequency units.

Notice that in the framework of social approach, the “lower” and the “upper” level definitions are conditional; these levels must be specified in a separate measurement procedure with using, for example, valence parameter. In general, one can establish the TLCSs as a spin-system operating with “spin-up” and “spin-down” states - see Fig. 1, cf.^44–46^.

In the framework of social paradigm an interaction between individuals in the network is realised by communication via messages. Hence, we can recognize each s-photon TLCS absorbs (or emits) as a message individual some way receives (or spreads) in the framework of the network model shown in Fig. 1. Thus, each social quantum represents some portion of socially actual information containing in one message. The frequency (social energy) of this quantum, ${\omega }_{{\rm{ph}}}$, that we suppose constant through the paper for all community, carries some certain context.

In the paper we consider Eq. (1) for a social community being in thermal equilibrium and characterized by some temperature, *T*, see Fig. 1. Initially, actors in a target network shown in Fig. 1 obtain some information from the outside. Then, this information field is “absorbed” and re-emitted by target network actors. Simultaneously there exist some people or information (message) exchange with huge “reservour network” community. This approach implies that our target social community is in permanent contact with a very large community of people (environment) that acts as a “reservour network” (RN)^89^. This mechanism provides thermalization for the joint coupled system of actors + information field and establishment of thermal equilibrium. Noticing that similar scenario is realized in condensed matter physics for thermalization of coupled matter-field states^78^. Below for our calculations we use the grand canonical ensemble approach with nonzero chemical potential.

As a result, the information field can be amplified or lost in the target subsystem. ${N}_{ph}=\langle {f}^{\dagger }f\rangle$ can be associated with an average number of messages (s-photons) inherent to the target network in Fig. 1. Thus, for thermal equilibrium of the system represented in Fig. 1 one can suppose that average number of messages and actors entering or leaving the target community remain constant. The role of the “reservour” community is to maintain an overall temperature *T* that, in fact, measures social activity of actors in communication. Notably, the model described in Fig. 1 is in line with the social cohesion framework described in^54^.

The temperature of the system can be defined, for instance, as in statistical physics by using free energy^89^. In this way it is possible to establish the essence of temperature for social systems, cf.^73^. In particular, Mimkes in^73^ exploits Lagrange principle to determine temperature and entropy contribution in behaviour characteristics obtained social systems. In our case we suggest an interpretation of the temperature effect that follows from thermodynamic characteristics of (ultracold) gases in statistical mechanics, cf.^90^. In particular, in “high” temperature limit the system in Fig. 1 represents a social network described by classical rules for “particles” representing actors and connections in the network, cf.^72^. There is not so much influence in the network community that can be characterized by some definite macroscopic polarization. The messages are exchanged chaotically, i.e. according to the principle of “ everyone with anyone “. As it is known in physics the quantum degeneracy of any paricle (or, even quasiparticle) gas occurs if we decrease the temperature (or, manipulate by the gas density)^90^. In this limit the overlapping of de Broglie waves associated with quantum properties of each particle in the gas ensemble appear. For the network community in Fig. 1 this situation can be explained through increasing the influence of actors on each other. In this limit quantum (wave)-like decision-making process dominates. The “macroscopic” effect of such an influence can be obtained by analyzing network conformation analysis, cf.^91^. In particular, we can recognise a “fit-get-rich” (FGR) phase when distinct phases of network community exist. In the limiting case of BEC large number of links attracted by the fittest node that represents a macroscopically large center of influence (social impact) in the network. The polarization of the community happens if there exist at least two such centers. This situation is typical for political blog networks cf.^92^. Thus, the temperature parameter is determined by network growing specifics (see e.g.^93^) and, as we can see below, by communication strength between the actors, cf.^94^.

Notably, our approach to network community analysis differs from one proposed in the framework of game theory, cf.^95,96^.

An absorption (emission) of socially actual information by *j*-th individual evokes excitation or, simply, polarization of *j*-th TLCS. In the framework of James-Lange theory of emotion, as example, such an event causes physiological arousal which is then interpreted as arousal related to individual emotion, cf.^50^. Thus, we can recognize an operator ${\sigma }_{-,j}={a}_{j}^{\dagger }{b}_{j}$ in Eq. (1) as an arousal of *j*-th individual.

The valence that represents a conjugate variable in the circumplex model of affect can be associated with “internal” degree of freedom, like a spin. In Eq. (1) we can recognize the valence as population imbalance operator ${\sigma }_{z,j}=\frac{1}{2}({b}_{j}^{\dagger }{b}_{j}-{a}_{j}^{\dagger }{a}_{j})$ for *j*-th individual.

The ${\sigma }_{z,j}$ admits three important averages. In particular, $\langle {\sigma }_{z,j}\rangle =-\,1/2$ if individual stay at (initial) mental state $|a\rangle$ after some (social) events of absorption or emission of photons. The state with $\langle {\sigma }_{z,j}\rangle =+\,1/2$ means changing of opinion by individual to the state $|b\rangle$. Value $\langle {\sigma }_{z,j}\rangle =0$ means that an individual remains undecided, or neutral on average.

The dynamical (Maxwell-Bloch like) equations obtained from Eq. (1) admit the integral of motion (cf.^97^)

2 $${\langle {\sigma }_{-,j}\rangle }^{2}+{\langle {\sigma }_{z,j}\rangle }^{2}=\frac{1}{4}.$$

Concerning collective emotions and decision-making problem it is fruitful to introduce collective variables

3 $$P=\frac{1}{N}\,\mathop{\sum }\limits_{j}^{N}\,\langle {\sigma }_{-,j}\rangle ,\,S=\frac{1}{N}\,\mathop{\sum }\limits_{j}^{N}\,\langle {\sigma }_{z,j}\rangle ,$$

which are polarization (*P*) and population imbalance (*S*) for close social community. Mathematically, *P* and *S* variables obey the same equation as (2).

**Figure 2:**
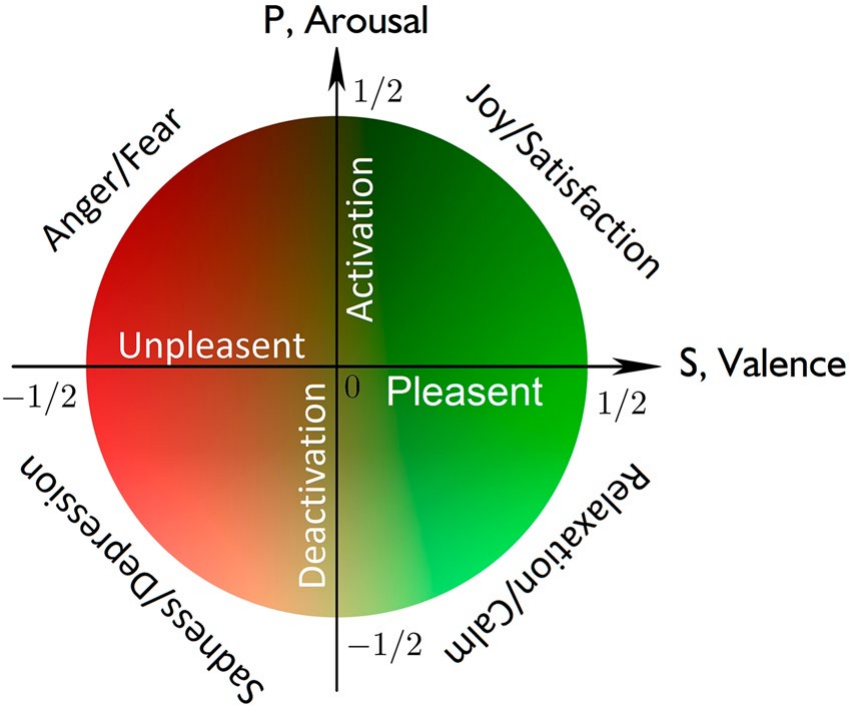
Arousal (*P*) - valence (*S*) plane representation of collective emotions caused by interaction of social community with information field. Green (red) color corresponds to positive (negative) valences which represent as pleasent (unpleasent) response, respectively. Dark to light colors transfer indicates transition from activation to deactivation arousal state, respectively. It is shown principal emotion states which are accessible for psychological and neirophysiological verification, cf.^49^.

Thus, we can recognize *P* and *S* variables as arousal and valence respectively, describing collective emotion states of some community being under the influence of information field, see Fig. 2 and cf.^50,51^. The arousal of some individual is typically measured by using some neirophysiological procedure, cf.^49^. The valence parameter can be measured by using appraisal evaluation. It can be connected with collective opinion formation in the network.

Then, the Hamiltonian (1) commutes with operator of total total excitation number

4 $${N}_{{\rm{ex}}}={f}^{\dagger }f+\mathop{\sum }\limits_{j}^{N}\,{\sigma }_{z,j}$$

representing the sum of photon number, i.e. number of messages and TLCS inversion that is a conserved quantity in our problem. Using definition (4) we can determine the normalized excitation density $\rho$ as:

5 $$\rho =\frac{\langle {N}_{{\rm{ex}}}\rangle }{N}={\lambda }^{2}+S,$$

where ${\lambda }^{2}={N}_{ph}/N$ is a normalized average photon number, i.e. a number of messages which evoke some information-oriented events in social community. Practically we are interested in different limits of Eq. (5).

So-called low excitation density limit implies ${\lambda }^{2}\to 0$ and TLCSs mostly populate their ground state $|a\rangle$, i.e. ${\sum }_{j}^{N}\,\langle {a}_{j}^{\dagger }{a}_{j}\rangle \approx N$^78^. In other words, individuals are still mostly unperturbed in this limiting case. The density $\rho$ can be estimated as

6 $$\rho \simeq -\,\mathrm{0.5.}$$

The saturation of the social system is achieved at excitation density $\rho =0$. For this limit lower and upper mental states are equally populated, i.e. ${\sum }_{j}^{N}\,\langle {b}_{j}^{\dagger }{b}_{j}\rangle \approx {\sum }_{j}^{N}\,\langle {a}_{j}^{\dagger }{a}_{j}\rangle =N/2$ and the valence $S=0$. In this case polarization *P* of community become maximal - see Fig. 2.

Finally, with densities $\rho \simeq 0.5$ an inversion of social system occurs, ${\sum }_{j}^{N}\,\langle {b}_{j}^{\dagger }{b}_{j}\rangle \gg {\sum }_{j}^{N}\,\langle {a}_{j}^{\dagger }{a}_{j}\rangle$. For ${\lambda }^{2}\gg 1$ the density of excitations $\rho$ becomes more photon-like. At the same time, nonlinear effects in matter-field interaction become important. This limit corresponds to social laser case.

### Thermodynamic approach to social network in s-field presence

At first, it is instructive to consider simple case when external information field is absent and all TLCSs are identical to each other possessing interaction with reservour network only. The collective valence parameter, *S*, simply reads as

7 $$S=-\,\frac{1}{2}\,\tanh \,(\frac{{\omega }_{{\rm{at}}}}{2{k}_{B}T}),$$

where *k*_*B*_ is Boltzmann constant, thereafter we took it equal to unity for simplicity.

The Eq. (7) implies that at the rest population of the (“ground”) state $|a\rangle$ is larger than (excited) one $|b\rangle$ by Boltzmann factor. In the limit of zero temperature $T=0$ when the system and interaction with reservour is”frozen” the *S* parameter is equal to −1/2.

In the opposite limit, $T\to \infty$, the *S* parameter approaches zero. It means that interaction with reservoir network results in forming some variety of opinions in the target community network. However, without external information field and information exchange it is impossible to achieve inversion in collective opinion formation.

In the presence of information field “subsystem” we apply the grand canonical ensemble to describe social system implies that the number of individuals *N* in observed (target) network community represents a non-conservative variable; it varies in accordance to perception and reaction on external information field that we quantize. As mentioned above, the properties of excitations of TLCS become important in this case.

The equation for *λ*-parameter in equilibrium may be derived from a variational thermodynamic approach, proposing grand canonical ensemble with chemical potential $\mu \ne 0$, cf.^77,78^. Skipping for simplicity some mathematical details, we obtain

8 $$\lambda {\tilde{\omega }}_{{\rm{ph}}}=\frac{\lambda }{N}\,\mathop{\sum }\limits_{j=1}^{N}\,\frac{{g}_{j}^{2}\,\tanh \,(\frac{1}{2T}\sqrt{{\tilde{\omega }}_{{\rm{at}},j}^{2}+4{g}_{j}^{2}{\lambda }^{2}})}{\sqrt{{\tilde{\omega }}_{{\rm{at}},j}^{2}+4{g}_{j}^{2}{\lambda }^{2}}},$$

possessing population inversion

9 $$\langle {\sigma }_{z,j}\rangle =-\,\frac{{\tilde{\omega }}_{{\rm{at}},j}\,\tanh \,(\frac{1}{2T}\sqrt{{\tilde{\omega }}_{{\rm{at}},j}^{2}+4{g}_{j}^{2}{\lambda }^{2}})}{2\sqrt{{\tilde{\omega }}_{{\rm{at}},j}^{2}+4{g}_{j}^{2}{\lambda }^{2}}},$$

where we made denotations ${\tilde{\omega }}_{ph}={\omega }_{ph}-\mu$, ${\tilde{\omega }}_{at,j}={\omega }_{at,j}-\mu$.

The *λ* is order parameter and plays crucial role in the phase-transition problem^76^. In particular, collective polarization (arousal) *P* is proportional on *λ*- parameter, i.e. $P\sim \lambda$.

The case of $\lambda \ne 0$ characterizes some coherent (condensed) solution for opinion formation. The existence of condensed solution leads to appearance of macroscopic polarization *P* of social (target) community that manifests social (coherent) energy release.

The value $\lambda =0$ defines so-called normal (non-condensed) phase state. Practically, it means that some socially important information characterized by *λ*-parameter lost in the target network due to variety of emotion states in it. For current information-oriented societies $\lambda =0$ value may be also interpreted as limiting case of total suppression of individuals' activity in communication due to interaction with environment at high enough social temperatures, see Fig. 1. There is no social energy release in this situation, socially positive or negative. Below we are interested in non-trivial solutions of (8) with $\lambda \ne 0$.

## Social SPT Model

### Basic equations

We consider solutions of (8) for describing heterogeneous social community decisions and emotional states. Obviously, humans are not identical regarding the information perception and communication. We take into account heterogeneous specifics of individuals, composing a social community with an analogy of inhomogeneous broadening known for artificial atom emission in material science^98,99^. To be more specific, we examine the model when only transition energy of TLCS varies, while for the coupling parameter we take ${g}_{j}\equiv g=const$. We suppose that ${\omega }_{at,j}={\omega }_{0}+{\xi }_{j}$, where ${\xi }_{j}$ is stochastic component characterizing *j*-th individual’s specific perception of external information; ${\omega }_{0}$ is an average energy spacing for individuals mental energy. We characterize the efficiency of interaction of each TLCS with socially actual information by detuning

10 $${\Delta }_{j}={\tilde{\omega }}_{ph}-{\tilde{\omega }}_{at,j}=\Delta -{\xi }_{j},$$

where $\Delta ={\omega }_{{\rm{ph}}}-{\omega }_{0}$ and ${\xi }_{j}$ are constant, and stochastic (fluctuated) parts of energy detuning, respectively.

In quantum theory the excitation probability of a two-level system for given nonzero *λ* is proportional to $4{g}^{2}{\lambda }^{2}/({\Delta }_{j}^{2}+4{g}^{2}{\lambda }^{2})$^100^. If ${\Delta }_{j}=0$, external information is in full resonance with internal transition frequency of mental energy of *j*-th individual. In this limit TLCS may be excited with maximal probability. For ${\Delta }_{j}^{2}\gg 4{g}^{2}{\lambda }^{2}$ the excitation probability of TLCS is vanishing and individuals’ motivation to change their ground mental state is very low. In this case information is completely out of resonance and socially irrelevant for the context of decision-making for individuals of chosen community.

Below we are interesting in the limit of large TLCS number in a target social system, $N\gg 1$. We suppose that random variable ${\xi }_{j}$ varies continuously according to Gaussian

11 $$f(\xi )=C\,\exp \,[\,-\,{\xi }^{2}/{\sigma }^{2}];$$

or Levy distribution laws

12 $$f(\xi )=C{Re}\,{\int }_{0}^{\infty }\,\exp \,[\,-\,2i\xi z/\sigma -{z}^{\alpha }]\,dz,$$

where *C* is a normalization constant, $\alpha \in (0,2]$. Parameter *σ* in (11), (12) defines broadening width and describes heterogeneity of a social system.

Noteworthy, Levy distribution in (12) is inherent to various applications to characterize social, economics and finance processes with large fluctuations^101^. Levy distribution (12) matches Gaussian one (11) for parameter $\alpha =2$. When $0 < \alpha  < 2$ Levy distribution (12) possesses so-called “heavy tales” for large $\xi$ that means growing of fluctuations when $\alpha \to 0$.

For large $N\gg 1$ one can replace the sum in (5) and (8) with integral introducing dimensionless frequencies ${\omega ^{\prime} }_{0}={\omega }_{0}/g$, ${\omega ^{\prime} }_{ph}={\omega }_{ph}/g$, $\xi ^{\prime} =\sigma \xi /g$, $\mu ^{\prime} =\mu /g$/. Replacing $\mu ^{\prime} \equiv {\omega }_{ph}+\mu ^{\prime}$ in (5) and (8) for Gaussian distribution (11) we get

13 $$\{\begin{array}{rcl}\Delta -\mu  & = & \frac{1}{\sqrt{\pi }}\,{\int }_{-\infty }^{\infty }\,\tfrac{\tanh \,[\tfrac{1}{2T}\sqrt{{(\sigma \xi -\mu )}^{2}+4{\lambda }^{2}}]{e}^{-{\xi }^{2}}d\xi }{\sqrt{{(\sigma \xi -\mu )}^{2}+4{\lambda }^{2}}};\\ \rho  & = & {\lambda }^{2}-\frac{1}{2\sqrt{\pi }}\,{\int }_{-\infty }^{\infty }\,\tfrac{(\sigma \xi -\mu )\,\tanh \,[\tfrac{1}{2T}\sqrt{{(\sigma \xi -\mu )}^{2}+4{\lambda }^{2}}]{e}^{-{\xi }^{2}}d\xi }{\sqrt{{(\sigma \xi -\mu )}^{2}+4{\lambda }^{2}}}.\end{array}$$

For simplicity of notation we dropped all the primes in (13). Similar set of equations may be obtained for Levy distribution (12).

### “Superradiance” of information field at zero temperature

We distinguish two limiting cases to analyze (13). First, we consider important zero temperature limit $T=0$ when the state is condensed or superfluid. As mentioned above, this state is socially favorable to observe quantum-like collective decision-making.

Analytic expressions for chemical potential *μ* may be derived from (13) in homogeneous limit assuming all TLCSs identical to each other, ${\omega }_{{\rm{at}},j}={\omega }_{0}$, i.e.

14 $${\mu }_{\pm }=\frac{1}{2}[{\omega }_{0}+{\omega }_{{\rm{ph}}}\pm {\omega }_{R}],$$

where we defined ${\omega }_{R}=\sqrt{{\Delta }^{2}-8\,(\rho -{\lambda }^{2})}$.

An occurrence of chemical potential branches for a social community can be understood as follows. The interaction of TLCSs with socially important (contextual) information field splits mental energy states. At steady state this reflects individuals’ opposing opinion to some resonant information context.

More generally, two branch mental energies can be associated with two mutually opposing communities, political parties, as example, perceiving information in opposing ways. The parameter ${\omega }_{R}$ characterizes “social energy distance” between these two major communities. Obviously, the ${\omega }_{R}$ behavior depends on how information is resonant and what is initial populations of TLCSs. The social energy distance vanishes under the resonance condition $\Delta =0$ and/or for $\rho ={\lambda }^{2}$. The latter requires positive density $\rho  > 0$, that corresponds to an initial inversion in TLCSs.

Below we focus mainly on a social community inherent to lower branch properties.

**Figure 3:**
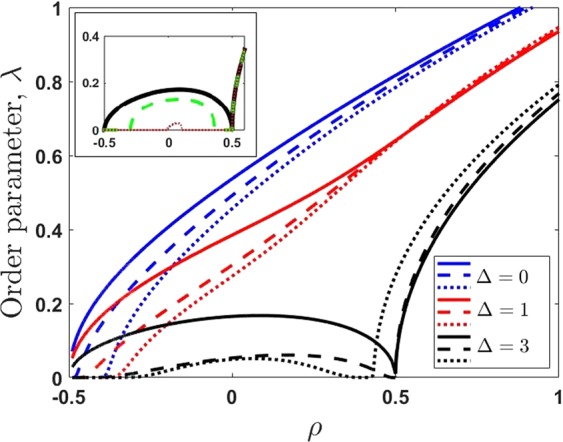
The order parameter *λ* vs. excitation density $\rho$ at zero temperature and for different values of detuning Δ. Solid lines correspond to homogeneous community limit with $\sigma =0$, dashed and dotted lines relay to Gaussian (${\sigma }_{G}=1$) and Levy (${\sigma }_{L}=1$) broadening, respectively; $\alpha =1$. The insert demonstrates the same dependence but for different temperatures. The black solid line corresponds to the limiting case of zero temperature, green (dashed) and red (dotted) lines corresponds to temperatures $T=1/8$ and $T=1/6$, respectively, given in dimension-less units. For the temperatures above the critical ${T}_{c}=1/5$ order parameter is $\lambda =0$.

Figure 3 represents the order parameter *λ* as a function of excitation density $\rho$. As it is seen from Fig. 3, the order parameter non-monotonically increases with $\rho$. The role of the heterogeneity (fluctuations) or broadening becomes important in low excitation limit $\rho =-\,0.5$. In particular, in a homogeneous case $\lambda =0$ and $\mu =\frac{\Delta }{2}-1$ for $\rho =-\,0.5$. However, for a heterogeneous (fluctuated) social system with $\sigma \ne 0$, $\mu \to -\,\infty$ at $\rho \to -\,0.5$.

Another practically important case is $\rho =0.5$, which corresponds to a population inversion. Interestingly, the order parameter is suppressed for $\Delta \ge 2$, when external information is out of exact resonance. It is possible to show that the switching between lower and upper mental energy branches occurs. In the general case, the heterogeneity of a social community leads to the washing out of the excited state, and relevant transitions become smoother; it is clearly seen from Fig. 3 at $\rho =0.5$.

At high social excitation densities of social system (for $\rho  > 0.5$), order parameter, *λ*, grows linearly and the number of photons becomes much larger than excitations in the system; this situation corresponds to the social laser limit when the inversion in mental state levels (valence) plays an essential role.

**Figure 4:**
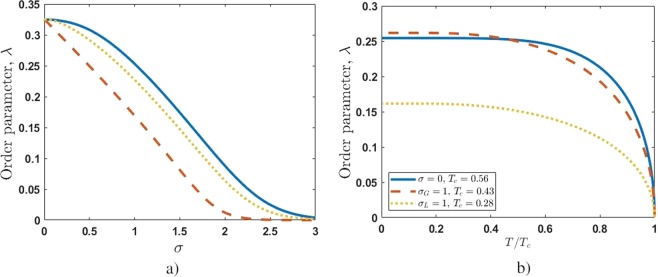
Order parameter *λ* vs. (**a**) - width of broadening *σ* at zero temperature, and (**b**) - normalized temperature *T*/*T*_*c*_ for $\rho =-\,0.3$ and $\Delta =0$. Solid line in (**a**) corresponds to Gaussian broadening, the dashed and dotted lines correspond to Levy broadening with $\alpha =1$ and $\alpha =1.5$, respectively. The $\sigma =0$ for (**b**) relays to the homogeneous case; the values ${\sigma }_{G}=1$ and ${\sigma }_{L}=1$ correspond to communities characterizing by Gaussian and Levy-broadening ($\alpha =1$), respectively.

Figure 4 reveals the impact of non-homogeneity in decision-making process performed by a social community. In Fig. 4 we plotted the dependencies of the order parameter, *λ*, as a function of *σ*, the width of Gaussian or Levy distribution. We take $\rho =-\,0.3$ and $\Delta =0$. Figure 4 demonstrates significant suppression of *λ* with increasing of *σ*. The suppression becomes dipper and happens earlier for the system possessing large fluctuations when parameter *α* vanishes (the curves for Gaussian and Levy distributions coincide at $\alpha =2$). The result obtained has a simple explanation: the coherent part of released energy is too low for a social community possessing broad variations in mental energy states or collective emotions.

### Superradiance properties at non-zero temperature

Now we examine the general case when the social temperature of a system is non-zero and a target network is in permanent contact with environment^73^. The main features of a social system in this case are determined by two effects acting oppositely. the information field tends to create some coherent polarization in the community which just means the synchronization effect for collective decision-making processes. Meanwhile, the thermal fluctuations evoked by random behavioral properties of individuals tends to suppress them. The Fig. 3 insert shows this behavior. In particular, the domain of non-zero order parameter *λ* vanishes when the temperature increase.

The dependences of order parameter *λ* as a function of normalized temperature *T*/*T*_*c*_ are represented in Fig. 4b. As clearly seen, there exists some critical temperature *T* = *T*_*c*_ when the system undergoes a second order continuous phase-transition to condensed or superfluid state. This state means the establishment of non-zero coherent macroscopic polarization in target network community of TLCSs which corresponds to superradiant phase with $\lambda  > 0$.

**Figure 5:**
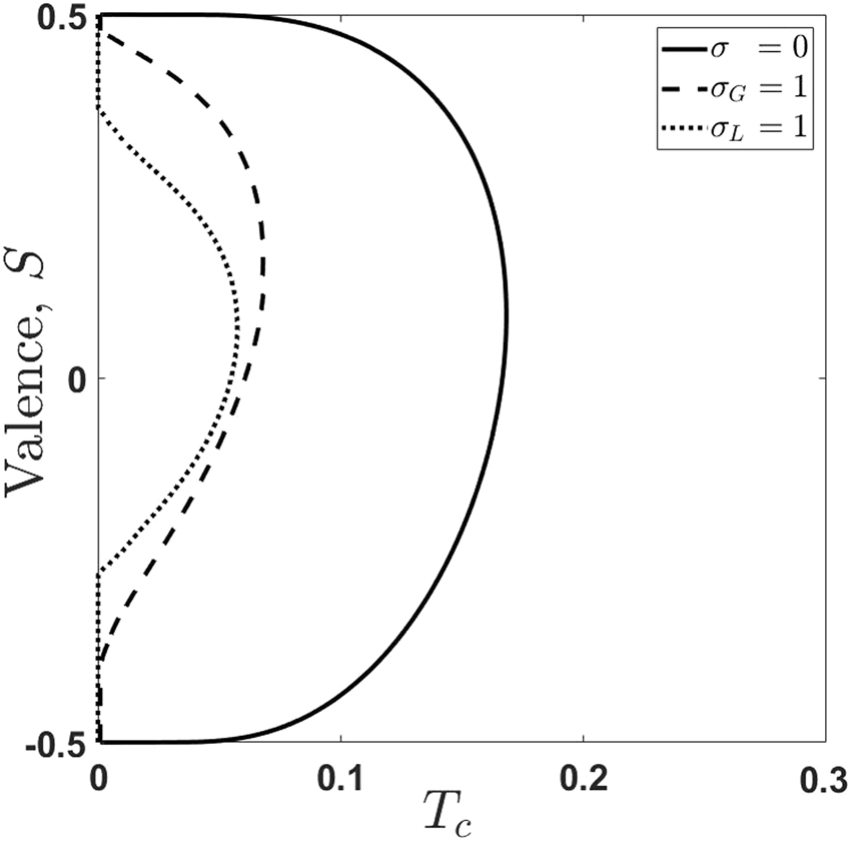
Population imbalance (valence) *S* vs. critical temperature, *T*_*c*_. The parameters are: $\lambda =0$ (phase boundary), $\Delta =3$. The $\sigma =0$ relays to the homogeneous case; the values ${\sigma }_{G}=1$ and ${\sigma }_{L}=1$ correspond to communities characterizing by Gaussian and Levy-broadening ($\alpha =1$), respectively.

Phase boundary (solution of Eq. (13) with $\lambda =0$) for the collective population imbalance (valence) is shown in Fig. 5. The value of Δ-parameter corresponds to the switching effect in valence, shown in Fig. 3 (black curves) for $T=0$. From Fig. 5 it is clearly seen that at high enough excitation densities the valence parameter, *S*, for target community changes from negative to positive values even at non-zero temperatures. Obviously, heterogeneity of social community affected this process negatively. The dependences with nonzero *λ*-parameter in Fig. 5 are located to the left of the corresponding boundary curves.

To understand the practical impact of critical temperature for a social community it is instructive to examine a simplified case of homogeneous system. Considering (13) at $T={T}_{c}$ and $\lambda =0$ for $\sigma \to 0$ we get

15 $${T}_{c\pm }=\frac{\Delta \pm \sqrt{\frac{{\Delta }^{2}}{4}-2\rho }}{2{\rm{arctanh}}\,[2\rho ]},$$

where ± signs correspond to upper and lower mental energy states (14), respectively.

Equation (15) has a very important impact for the behavioral quantification of social systems. The social temperature of phase-transition in information oriented societies is completely determined by excitation density $\rho$ and parameter Δ, related to the context and collective emotions in the social community.

The behaviour of *T*_*c*_ on $\rho$ can be obtained by solving Eq. (13). In particular, infinite critical temperature ${T}_{c}\to \infty$ corresponds to the limit of $\rho \to 0$ that implies saturation of mental states, i.e. population of upper and lower states become equal to each other. This phenomenon has a clear explanation. The separation of society in two equal halves significantly resists and complicates any collective decision-making suitable for the whole system.

## Relevance to the Other Models with Social Impact

In this section we consider the connection of our model with some other models known in the framework of the social impact paradigm^40–43,45–48,50^. At first, we rewrite Eq. (1) in more general form as ($\hslash =1$)

16 $$H={\omega }_{{\rm{ph}}}{f}^{\dagger }f+\mathop{\sum }\limits_{j}^{N}\,{\omega }_{{\rm{at}},j}{\sigma }_{z,j}+\frac{1}{2\sqrt{N}}\,\mathop{\sum }\limits_{j}^{N}\,{g}_{j}{\sigma }_{x,j}({f}^{\dagger }+f),$$

where ${\sigma }_{z,j}$, and ${\sigma }_{x,j}$ are Pauli spin operators. The Heisenberg equation for the field operator *f* is

17 $$i\frac{df}{dt}={\omega }_{{\rm{ph}}}f+\frac{1}{2\sqrt{N}}\,\mathop{\sum }\limits_{j}^{N}\,{g}_{j}{\sigma }_{x,j}.$$

Now we suppose that the information field is already established in the social community. Practically, this limit can be realized within large time scale when the social community undergoes the action of information field in the state induced by large information reservoir, representing a huge amount of mass-media. In quantum physics this approach is sometimes referred as a bad cavity limit when cavity field may be illuminated^102,103^. Setting in Eq. (17) $\frac{df}{dt}=0$ we obtain

18 $$f=-\,\frac{1}{2{\omega }_{{\rm{ph}}}\sqrt{N}}\,\mathop{\sum }\limits_{j}^{N}\,{g}_{j}{\sigma }_{x,j}.$$

Substituting Eq. (18) into Eq. (16) one can get

19 $$H=\mathop{\sum }\limits_{j}^{N}\,{\omega }_{{\rm{at}},j}{\sigma }_{z,j}-\frac{1}{4{\omega }_{{\rm{ph}}}N}\,\mathop{\sum }\limits_{i,j}^{N}\,{g}_{i}{g}_{j}{\sigma }_{x,i}{\sigma }_{x,j}.$$

Equation (19) exactly corresponds to familiar Ising Hamiltonian used in cellular automata modeling of two-state opinion formation^45,46^. In particular, the correspondence becomes more evident if we define

20 $${g}_{i}{g}_{j}/{\omega }_{{\rm{ph}}}={s}_{j}/{r}_{ij}$$

for coupling coefficients ${g}_{i}{g}_{j}/{\omega }_{{\rm{ph}}}$ characterizing our model. An ability to influe of *j*-th individual to the others is defined by positive parameter *s*_*j*_ and *r*_*ij*_ is some increasing function that defines social distance in the opinion formation model.

Thus, Eq. (20) establishes a direct link between measured parameters of our model and the ones of social impact models that can be used for practical purposes. Such measurements may be performed by using current technologies of community detection in social networks^104^.

However, it is important that for Eq. (19) we don’t need to use the grand canonical ensemble approach to describe our social system; the number of individuals *N* is fixed now^89^. Noteworthy, the information field elimination implied by cellular automata model, see Eq. (18), leads to complete loosing of the important information about collective emotions. Contrary, the model that we offer in the paper focuses on the “interaction” of individuals with external information field revealing collective emotions.

## Conclusion

Let us briefly summarize the results obtained. In the paper we focus on the collective decision-making and collective emotions problems for information oriented social communities when the mass-media and Internet resources play an essential role in frequent communication for individuals. Our work is inspired by recent publication^60^ that relays on the laser effect occurrence in a social community possessing quantum-like discrete mental states in respect of their decision-making problem.

As we have shown, the social laser represents only one possible mechanism to release coherent social energy. It requires a strong external information pump and inversion in mental states characterized by the valence parameter. On the contrary, collective decision-making may be achieved in an information oriented social system without inversion at all due to the occurrence of the second order continuous phase-transition to some superradiant state, which is relevant to superfluid state of a social community. Strictly speaking such a state in modern sociology can be interpreted as social cohesion state when “ the level of community is, for example the shared loyalties, mutual moral support, social capital, strong social bonds, trust, social environment, formal/informal control, overlap of individuals’ friendship networks, pressures for conformity and caring, civic society, reciprocal loyalty and solidarity, strength of social relations, shared values, common goals, moral behaviour and norms, values of rewards in groups, and process performance and goal attainment”^54^.

In the paper we give a clear explanation how interaction with information field helps to describe collective emotional states. For that we have introduced new variables which are arousal (macroscopic polarization) and valence (macroscopic population imbalance). More precisely, we refer to some target network community, which is in close interaction with “reservour” (large network community) possessing infinite degree of freedom. We used the grand canonical ensemble approach known in statistical physics taking into account the variation of number of individuals in a community that represents a small part of a huge social system or a social reservour. In equilibrium the whole social system, i.e. target system + reservour, has some social temperature *T* that characterizes social activity of individuals.

In general, as we have shown, the interaction with the information field results in formation of two large groups of individuals with different beliefs and decisions. The social distance between them, which is Rabi splitting, characterizes relevant decisions which correspond to transitions on *μ*_±_ frequencies. In our model we take into account two characteristics which are relevant to random properties of individuals’ community. The first one is the social temperature that characterizes behavioral randomness, and the second one is inherent to mental energy broadening, which determines heterogeneous specifics of the chosen community. In particular, we examine the influence of Gaussian and Levy broadening to decision-making process. We have shown that statistical fluctuations suppress collective (coherent) character of this process. However, there exists some critical temperature of a social system when the phase-transition to some coherent state of information field occurs and macroscopic polarization of target community appears. It is important that social super-radiant state directly depends on collective emotions appearing in community under decision-making process.

Notably, the superradiant state implies coherent social energy release (lasing) without any inversion in TLCS population. The results obtained are clearly interpreted in the framework of early models of social impact. In particular, the cellular automata social model of two-state opinion formation is valid if some contextual information field is already established in a social community. However, collective emotions in this case are still hidden due to the elimination of information field (polarization) that characterizes arousal in behavioural properties of a society.

Our results are likely to have a new promising practical impact. Even if the social network system is out of thermal equilibrium, the arousal and valence variables proposed adequately describe collective emotion states due to some dynamical properties. However, this problem requires separate analysis and some other approaches known in laser physics, cf.^57^. Checking them in social networks seems a challenging problem for further research.
